# Physical activity and metabolic health in chronic kidney disease: a cross-sectional study

**DOI:** 10.1186/s12882-016-0400-x

**Published:** 2016-11-22

**Authors:** Wilson Bowlby, Leila R. Zelnick, Connor Henry, Jonathan Himmelfarb, Steven E. Kahn, Bryan Kestenbaum, Cassianne Robinson-Cohen, Kristina M. Utzschneider, Ian H. de Boer

**Affiliations:** 1 University of Washington School of Medicine, Seattle, WA USA; 2 Division of Nephrology and Kidney Research Institute, University of Washington, Seattle, WA USA; 3 VA Puget Sound Health Care System, Seattle, WA USA; 4 Division of Metabolism, Endocrinology, and Nutrition, University of Washington, Seattle, WA USA

**Keywords:** Chronic kidney disease, Physical activity, Metabolism, Insulin resistance, Obesity, Triglycerides

## Abstract

**Background:**

Patients with chronic kidney disease (CKD) are at high risk of progression to end stage renal disease and cardiovascular events. Physical activity may reduce these risks by improving metabolic health. We tested associations of physical activity with central components of metabolic health among people with moderate-severe non-diabetic CKD.

**Methods:**

We performed a cross-sectional study of 47 people with CKD (estimated GFR <60 ml/min/1.73 m^2^) and 29 healthy control subjects. Accelerometry was used to measured physical activity over 7 days, the hyperinsulinemic-euglycemic clamp was used to measure insulin sensitivity, and DXA was used to measured fat mass. We tested associations of physical activity with insulin sensitivity, fat mass, blood pressure, serum lipid concentrations, and serum high sensitivity C-reactive protein concentration using multivariable linear regression, adjusting for possible confounding factors.

**Results:**

Participants with CKD were less active than participants without CKD (mean (SD) 468.1 (233.1) versus 662.3 (292.5) counts per minute) and had lower insulin sensitivity (4.1 (2.1) versus 5.2 (2.0 (mg/min)/(μU/mL)), higher fat mass (32.0 (11.4) versus 29.4 (14.8) kg), and higher triglyceride concentrations (153.2 (91.6) versus 99.6 (66.8) mg/dL). With adjustment for demographics, comorbidity, medications, and estimated GFR, each two-fold higher level of physical activity was associated with a 0.9 (mg/min)/(μU/mL) higher insulin sensitivity (95% CI 0.2, 1.5, *p* = 0.006), an 8.0 kg lower fat mass (−12.9, −3.1, *p* = 0.001), and a 37.9 mg/dL lower triglyceride concentration (−71.9, −3.9, *p* = 0.03). Associations of physical activity with insulin sensitivity and triglycerides did not differ significantly by CKD status (*p*-values for interaction >0.3).

**Conclusions:**

Greater physical activity is associated with multiple manifestations of metabolic health among people with moderate-severe CKD.

**Electronic supplementary material:**

The online version of this article (doi:10.1186/s12882-016-0400-x) contains supplementary material, which is available to authorized users.

## Background

People with chronic kidney disease (CKD) are at high risk of cardiovascular disease (CVD) [1]. Reduced sensitivity to the actions of insulin, i.e. insulin resistance, is one mechanism through which CKD may promote CVD [2]. Patients with CKD are often insulin resistant [3–5]. Insulin resistance is a central component of the metabolic syndrome, an adverse metabolic milieu that includes obesity, hyperglycemia, dyslipidemia, and hypertension and is associated with activation of the renin-angiotensin-aldosterone system, oxidative stress, inflammation, and endothelial dysfunction [2, 6, 7]. Insulin resistance and these interrelated metabolic abnormalities have been associated with increased risks of atherosclerosis and cardiovascular events as well as progression of CKD to end stage renal disease [8–11].

Interventions that aim to increase physical activity are promising approaches to improve metabolic health and clinical outcomes in CKD. In people without CKD, physical activity reduces adiposity and mitigates the metabolic syndrome [12, 13]. In the CKD population, physical activity is often low, and greater higher physical activity is associated decreased risks of CKD progression [14] and mortality [15]. However, few studies have assessed the metabolic pathways through which physical activity may lead to improved outcomes in CKD.

We quantified physical activity using accelerometers in a cross-sectional study of people with and without moderate-severe CKD, none of whom had clinical diabetes [4]. We examined relationships of objectively measured physical activity with insulin sensitivity quantified using the gold standard hyperinsulinemic-euglycemic clamp [16] and with related measures of metabolic health. We hypothesized that higher levels of physical activity would be associated with greater insulin sensitivity, reduced adiposity, an improved lipid profile, lower blood pressure, and lower levels of systemic inflammation.

## Methods

### Study population

The Study of Glucose and Insulin in Renal Disease (SUGAR) is a cross-sectional study of glucose and insulin metabolism among individuals who have moderate-severe nondiabetic CKD and healthy control individuals who do not have kidney disease [4]. Participants were recruited from nephrology and primary care clinics associated with the University of Washington and neighboring institutions in Seattle, Washington, from 2011 to 2104 (Additional file 1: Figure S1). SUGAR enrolled 59 participants with non-diabetic stage 3–5 CKD (estimated glomerular filtration rate <60 mL/min/1.73 m^2^ not treated with dialysis) and 39 control subjects (estimated glomerular filtration rate >60 mL/min/1.73 m^2^) with comparable distributions of age, sex, and race. Exclusion criteria for both groups included age <18 years, a clinical diagnosis of diabetes, maintenance dialysis or fistula in place, history of kidney transplantation, use of medications known to reduce insulin sensitivity (including corticosteroids and immunosuppressants), fasting serum glucose ≥126 mg/dL, and hemoglobin <10 g/dL. For this study, we included 76 SUGAR participants who collected accelerometry data for ≥480 min on each of three or more days, excluding 12 who did not perform accelerometry and 10 whose accelerometry data were insufficient.

### Accelerometry

We quantified physical activity using the ActiGraph GT3X. The GT3X measures movement in three planes (x-axis, y-axis, z-axis). Movements in all three planes were summed for each 60-s time period (1-min epoch) to generate movement in counts per minute (CPM). Study participants were asked to wear an accelerometer at their waist at all times through a consecutive 7-day period, including weekdays and weekend days, removing the accelerometer only during sleep and water-based activities. ActiLife v5.10.0 was used to upload, clean, and analyze collected data.

Accelerometry data reduction and analysis were based on prior reports [17, 18]. Participants were included in analyses if they wore an accelerometer for at least 8 h (approximately 60% of wake time) on at least 3 days (approximately half of the requested number of wear days). Non-wear periods were defined as intervals of at least 60 min during which no more than 2 min registered greater than zero CPM and no single minute registered >100 CPM. For each sufficient participant-day, CPM was calculated as the total number of movement counts divided by total wear time. For each participant with at least three qualified wear days, we calculated mean CPM, the primary exposure for this study, as the simple mean of CPM for all days with sufficient data. As secondary exposures, we also examined proportions of time spent active (i.e. non-sedentary) and in moderate-vigorous physical activity [18]. For each minute of wear time, ≤59 CPM was classified as sedentary, and >59 CPM was classified as active.

Participants also completed the Human Activity Profile (HAP) as a parallel assessment of self-reported physical activity, and the adjusted HAP score was used as a third exposure [19].

### Metabolic health

Insulin sensitivity was measured using by hyperinsulinemic-euglycemic clamp [4], based on the method of DeFronzo et al. [16]. Participants were admitted to the University of Washington Clinical Research Center after an overnight fast. An insulin infusion was administered as a prime (160 mU/m^2^/min for 5 min) followed by a constant infusion (80 mU/m^2^/min). Blood glucose was measured every 5 min, and a variable rate of unlabeled 20% dextrose was infused to maintain blood glucose at approximately 90 mg/dL. Beginning 120–150 min after initiation of the insulin infusion, the dextrose infusion rate was held constant for 30 min, over which three steady-state plasma samples were obtained 15 min apart. Plasma concentrations of insulin and glucose were measured by two site immune-enzymometric assay (Tosoh 2000 auto-analyzer) and the glucose hexokinase method (Roche Module P Chemistry autoanalyzer), respectively. The dextrose concentration of the infusate was similarly quantified. Insulin sensitivity (SI) was calculated as (glucose disposal rate adjusted for drift in plasma glucose x concentration of infused glucose)/(insulin concentration at steady state – fasting insulin concentration) [16]. We calculated HOMA-IR from fasting insulin and glucose concentrations as an alternate assessment of insulin resistance [20].

Body composition (fat mass and fat-free mass) was measured by DXA (GE Lunar or Prodigy and iDXA, EnCore Software versions 12.3 and 14.1). Quality assurance procedures were followed on a daily basis as the calibration block and spine phantom were scanned and the analyzed readings were ensured to be within 1.5% of the actual measured quantities.

Blood pressure (BP) was measured on two occasions (immediately prior to the hyperinsulinemic-euglycemic clamp and approximately 1 week later). On each occasion, BP was measured three times, 5 min apart, in the seated position. The mean values of the final two measurements from each visit were used for analysis.

High sensitivity C-reactive protein (CRP), total cholesterol, HDL cholesterol, and trigylcerides were measured on the Beckman DXC600 automated chemistry platform systems. SYNCHRON systems quantitatively measures C-reactive protein, total cholesterol, HDL cholesterol, and triglycerides in human serum or plasma by rate turbidity. For all assays, interassay coefficients of variation were less than 5%.

### Covariates

Demographics and medical history were self-reported. Prevalent cardiovascular disease was defined as a physician diagnosis of myocardial infarction, stroke, resuscitated cardiac arrest, or heart failure or a history of coronary or cerebral revascularization [4]. Medications were ascertained by the inventory method. Serum creatinine and cystatin C were measured in fasting serum collected immediately prior to the clamp using a Beckman DxC automated chemistry analyzer. Creatinine concentration was traced to isotope dilution mass spectrometry values and cystatin C concentration was calibrated to ERM-DA471/IFCC. GFR was estimated from creatinine and cystatin C concentrations using the CKD-EPI formula [21]. Urine albumin was measured using a turbidimetric method on a Beckman Dxc automated chemistry analyzer (interassay coefficient of variation 0.8–1.7%).

### Statistical analysis

Participant characteristics and measures of metabolic health were summarized by tertiles of physical activity. Scatterplots with linear regression lines were used to graphically examine associations of physical activity with metabolic health outcomes, stratified by CKD status. Multivariable linear regression was used to test associations of every doubling of physical activity with metabolic health outcomes, assessing the full study population together for primary analyses. A series of nested models was created accounting for potential confounding covariates. The first model adjusted for demographic data: age (continuous variable), sex, and race (white/black/other). The second model additionally adjusted for cardiovascular disease and eGFR (continuous variable). The third model additionally adjusted for fat mass (continuous variable), which was considered either a confounder or mediator of the associations of interest. Medications were included in models if they directly affect the metabolic variable being assessed. Specifically, lipid-lowering medication classes were included as covariates when modeling triglycerides or HDL cholesterol as an outcome and antihypertensive medications (yes/no categorical variable plus number of medications modeled as a continuous variable) when modeling BP as an outcome. Missing data were multiply imputed and combined using Rubin’s rules. Interaction terms were used to test for differences in associations by CKD status. Two-way interactions were tested using multiplicative terms in the regression models with Wald tests to evaluate significance. All analyses were performed using Stata 14.0 and R version 3.2.1 [22]. Study investigators and staff were not blinded to CKD status, accelerometer data, or measures of metabolic health.

## Results

**Fig. 1:**
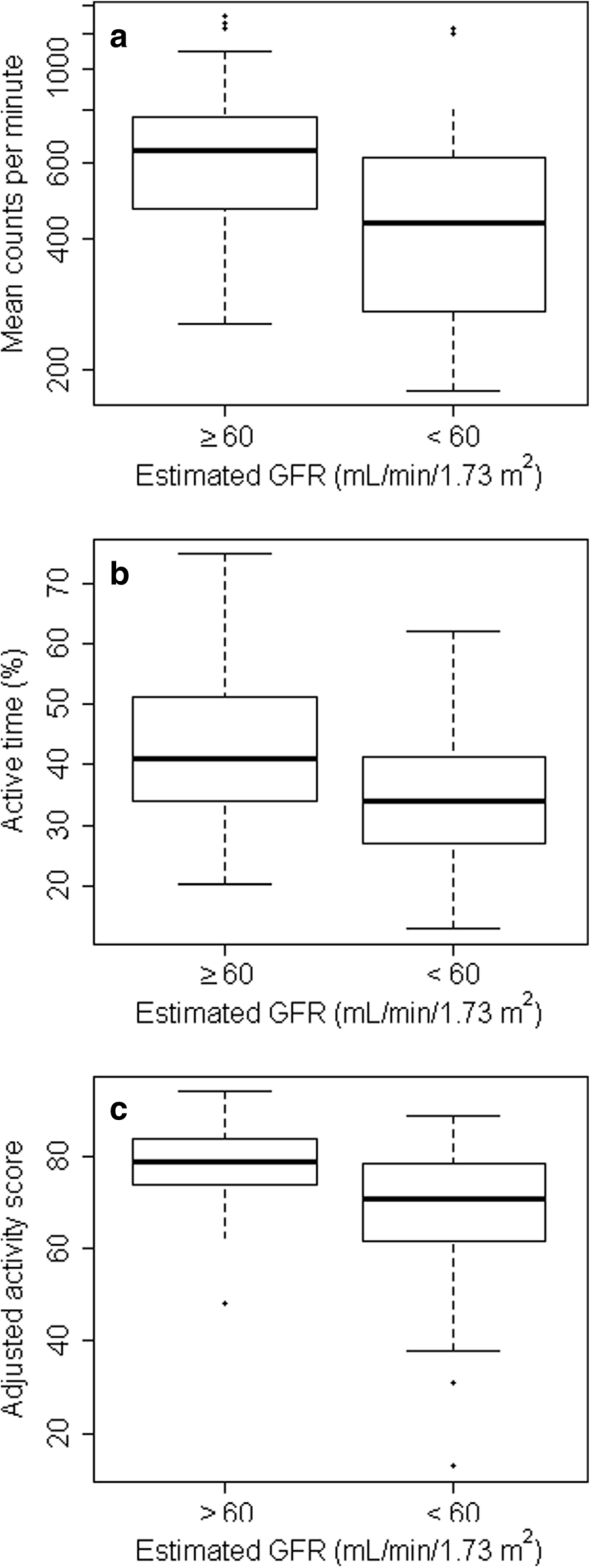
Physical activity among participants with and without CKD. Boxplots compare the physical activity of non-CKD vs CKD participants. Physical activity was quantified as accelerometry counts per minute (panel **a**), accelerometry active time (panel **b**), or human activity profile adjusted activity score (panel **c**). Box plots display median with the 25 and 75th percentiles, with participants outside 1.5 times the IQR noted as data points

Of the 76 SUGAR participants included in this study, mean age was 62.6 years, 44.7% were female, and race was self-reported as black for 14.5% and Asian or Pacific Islander for 5.3% (Table 1). 47 participants had CKD and 29 did not. Characteristics of the 76 SUGAR participants included in this analysis were similar to those of the 22 SUGAR participants excluded from this analysis (Additional file 1: Table S1). Participants with greater physical activity tended to be younger, weighed less, and had a higher eGFR (Table 1). Among participants with CKD, mean eGFR was 38.7 mL/min/1.73 m^2^ and median AER was 39.2 mg/24 h (Additional file 1: Table S2). Participants with CKD were less physically active than participants without CKD (Fig. 1 and Additional file 1: Table S2).

**Table 1:** Characteristics of SUGAR participants, by physical activity

	Physical activity		
	Tertile 1 (≤375 CPM)	Tertile 2 (375 < CPM ≤ 631)	Tertile 3 (> 631 CPM)
N	25	25	26
Demographics:			
Age (years)	69.5 (10.5)	63.0 (12.5)	58.1 (11.2)
Female sex	9 (36)	11 (44)	14 (54)
Race			
White	20 (80)	21 (84)	20 (77)
Black	4 (16)	2 (8)	5 (19)
Other	1 (4)	2 (8)	1 (4)
Medical history & lifestyle:			
Cardiovascular disease	13 (52)	2 (8)	3 (12)
Current smoking	5 (20)	1 (4)	5 (19)
Physical activity (adjusted activity score), median	67.0 (57.0–73.0)	76.0 (71.0–82.0)	78.5 (74.5–82.8)
Average Sedentary vs Active Time (%)	74.5	63.0	49.8
Medication use:			
Any antihypertensive medication	20 (80)	16 (64)	16 (62)
Number of antihypertensive medications	2.3 (1.5)	1.5 (1.8)	1.3 (1.5)
Any lipid-lowering medication	9 (36)	8 (32)	5 (19)
Statin	9 (36)	8 (32)	4 (15)
Fibrate	3 (12)	1 (4)	1 (4)
Niacin	2 (8)	0 (0)	1 (4)
Physical characteristics:			
Height (cm)	172.5 (9.3)	170.5 (10.0)	173.5 (11.8)
Weight (kg)	94.9 (20.1)	85.8 (15.4)	80.1 (20.8)
Fat mass (kg)	35.7 (13.3)	31.2 (12.4)	26.3 (11.3)
BMI (kg/m^2^)	31.8 (6.2)	29.6 (5.3)	26.4 (5.3)
Systolic blood pressure (mm Hg)	135.1 (14.4)	130.7 (12.1)	124.3 (17.2)
Diastolic blood pressure (mm Hg)	78.0 (9.6)	79.9 (8.4)	77.4 (11.3)
Laboratory data:			
Serum creatinine (mg/dL), median	1.6 (1.2–1.9)	1.4 (1.0–1.8)	1.0 (0.8–1.5)
Serum cystatin C (mg/L), median	1.6 (1.1–2.0)	1.2 (1.0–1.6)	1.0 (0.8–1.4)
Estimated GFR (mL/min/1.73 m^2^)	44.7 (21.7)	54.8 (28.4)	71.6 (26.5)
eGFR < 60 mL/min.1.73 m^2^	21 (84)	15 (60)	11 (42)
Urine AER, (mg/24 h) median	24.6 (8.2–137.4)	9.7 (5.3–91.7)	8.9 (5.6–95.4)

Mean (SD) presented for continuous variables, and median (IQR) as noted; N (%) presented for all categorical variables. Values were missing for fat mass (*N* = 4) and Urine AER (*N* = 3). *CPM* counts per minute, *GFR* glomerular flow rate, *AER* Albumin excretion rate

Physical activity was positively correlated with insulin sensitivity and serum HDL cholesterol concentration and negatively correlated with fasting insulin concentration, HOMA-IR, fat mass, SBP, serum CRP and triglyceride concentrations (Table 2). Adjusting for demographics, cardiovascular disease, and eGFR (Model 2) each doubling of CPM was associated with a 0.9 (mg/min)/(μU/mL) higher insulin sensitivity (95% CI 0.2,1.5 (mg/min)/(μU/mL), *p* = 0.006), an 8 kg lower fat mass (95% CI −12.9, −3.1 kg, *p* = 0.001), and a 37.9 mg/dL lower triglyceride concentration (95% CI −71.9, −3.9 mg/dL, *p* = 0.03) (Table 3). With further adjustment for fat mass (Model 3), the associations of physical activity with insulin sensitivity and triglycerides were modestly attenuated, and only the association with insulin sensitivity remained statistically significant (0.7 (mg/min)/(μU/mL) per doubling of CPM, 95% CI 0, 1.4 (mg/min)/(μU/mL), *p* = 0.04). Associations of physical activity with SBP, HDL cholesterol, and CRP were not significant in adjusted analyses. When mean CPM was replaced with active time (versus sedentary time) or moderate-vigorous as the exposure of interest, the results were similar with no significant changes to interpretation (Additional file 1: Tables S3–S5). When mean CPM was replaced with Adjusted Human Activity Profile score (HAP) as the primary exposure of interest, associations with insulin sensitivity and triglycerides were weaker (Additional file 1: Table S6).

**Table 2:** Metabolic health measurements in SUGAR, by level of physical activity

	Physical activity			
	Tertile 1 (≤ 375 CPM)	Tertile 2 (375 < CPM ≤ 631)	Tertile 3 (> 631 CPM)	*p*-value
Insulin sensitivity (mg/min)/(μU/mL)	3.8 (1.5)	4.5 (2.8)	5.2 (1.8)	0.009
Fasting glucose (mg/dL)	103.2 (8.9)	103.2 (10.0)	98.8 (8.6)	0.12
Fasting insulin (uU/mL)	11.1 (5.4)	9.7 (5.6)	6.6 (4.6)	0.004
HOMA-IR	2.8 (1.9)	2.4 (1.6)	1.5 (1.1)	0.003
Fat mass (kg)	35.7 (13.3)	31.2 (12.4)	26.3 (11.3)	0.03
BMI (kg/m^2^)	31.8 (6.2)	29.6 (5.3)	26.4 (5.3)	0.002
CRP (mg/dL), median	0.2 (0.2–0.4)	0.2 (0.1–0.6)	0.1 (0.1–0.3)	0.07
HDL (mg/dL)	52.2 (28.0)	50.6 (14.4)	61.2 (18.4)	0.06
Triglycerides (mg/dL), median	135.0 (114.0–191.0)	120.0 (73.0–163.0)	85.0 (58.5–120.2)	0.007
Systolic blood pressure (mm Hg)	135.1 (14.4)	130.7 (12.1)	124.3 (17.2)	0.046
Diastolic blood pressure (mm Hg)	78.0 (9.6)	79.9 (8.4)	77.4 (11.3)	0.61

Entries are mean (SD), except as noted. Some values were missing for Matsuda (*N* = 1), CRPH (*N* = 1) and Fat Mass (*N* = 4). *CPM* counts per minute, *CRP* high sensitivity C-reactive protein, *BMI* body mass index, *HDL* high density lipoprotein cholesterol

**Table 3:** Associations of physical activity with metabolic health outcomes in SUGAR

	Unadjusted	Model 1	Model 2	Model 3
Outcomes:				
Insulin sensitivity (mg/min)/(μU/mL)	1.0 (0.4, 1.5)	0.8 (0.2, 1.4)	0.9 (0.2, 1.5)	0.7 (0.0, 1.4)
*P Value*	0.0003	0.009	0.006	0.04
Fat mass (kg)	−6.3 (−10.3, −2.2)	−8.2 (−12.8, −3.6)	−8.0 (−12.9, −3.1)	NA
*P Value*	0.002	0.0005	0.001	NA
BMI (kg/m^2^)	−2.7 (−4.6, −0.8)	−3.6 (−5.8, −1.5)	−3.6 (−5.9, −1.3)	NA
*P Value*	0.005	0.0007	0.002	NA
CRP (% difference)	−33 (−51, −7)	−32 (−50, −6)	−24 (−48, 13)	−5 (−37, 43)
*P Value*	0.01	0.02	0.17	0.83
HDL (mg/dL)	5.6 (−2.3, 13.5)	6.6 (−2.0, 15.3)	6.8 (−0.8, 14.4)	5.5 (−1.5, 12.5)
*P Value*	0.17	0.13	0.08	0.12
Triglycerides (mg/dL)	−39.9 (−72.4, −7.5)	−45.0 (−76.2, −13.9)	−37.9 (−71.9, −3.9)	−22.7 (−57.0, 11.6)
*P Value*	0.02	0.005	0.03	0.20
Systolic BP (mm Hg)	−5.8 (−10.7, −1.0)	−4.5 (−9.1, 0.0)	−3.0 (−8.0, 2.0)	0.2 (−4.6, 5.1)
*P Value*	0.02	0.053	0.24	0.93
Diastolic BP (mm Hg)	0.3 (−2.9, 3.5)	−1.2 (−4.1, 1.7)	−1.1 (−4.2, 1.9)	−0.5 (−3.5, 2.6)
*P Value*	0.87	0.42	0.47	0.77

Entries are the difference (95% CI) in the outcome associated with a doubling in accelerometry mean counts per minute. Model 1 adjusts for age, sex, and race (white/black/other). Model 2 additionally adjusts for cardiovascular disease and eGFR. For HDL and triglycerides outcomes, Model 2 additionally adjusts for statins, fibrates, and niacin medications; for BP outcomes, Model 2 additionally adjusts for the number of hypertension medications. Model 3 adjusts for Model 2 variables plus fat mass. *BMI* body mass index, *CRP* C-reactive protein, *HDL* high density lipoprotein cholesterol, *BP* blood pressure

**Fig. 2:**
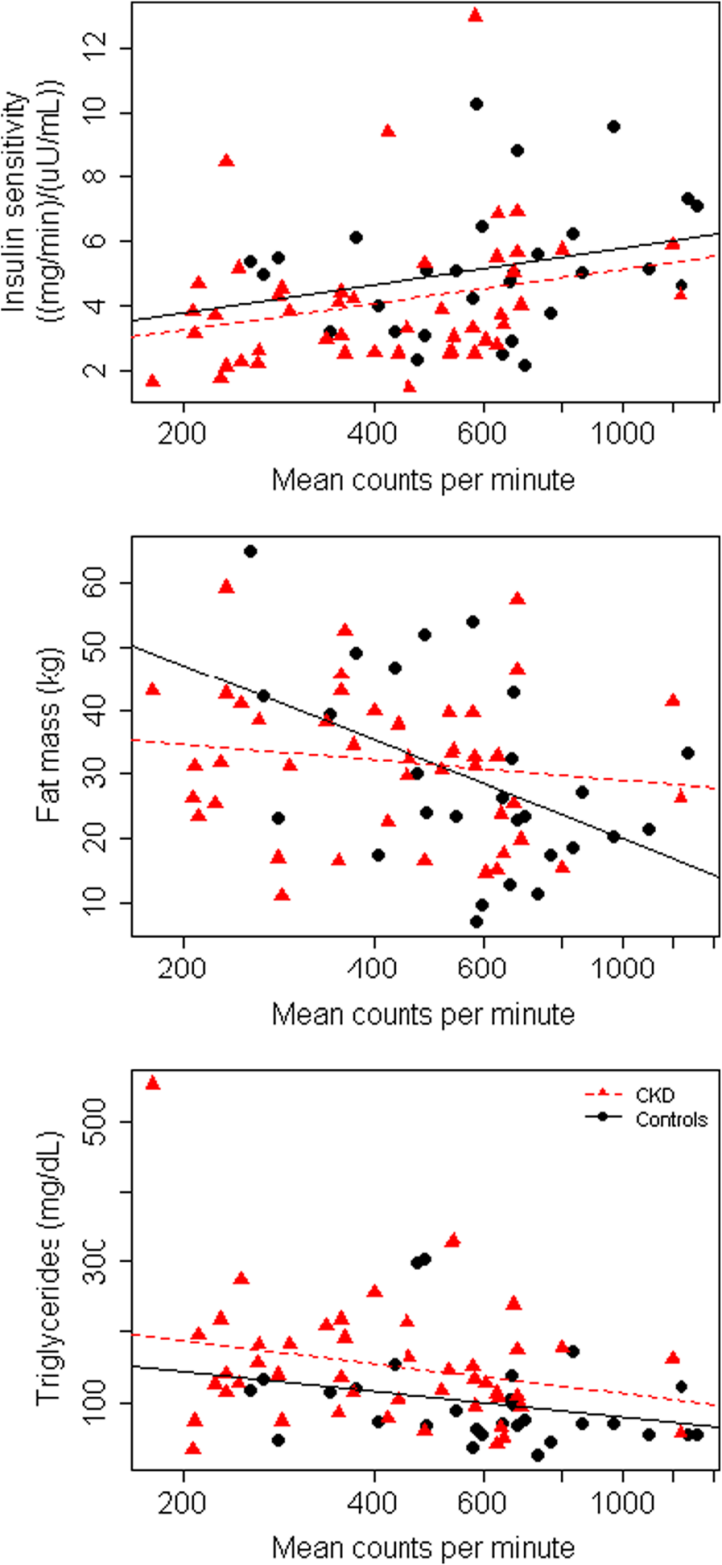
Correlations of physical activity measured by accelerometry with metrics of metabolic health. Scatter plots with linear regression lines stratified by CKD status. Controls are indicated in *red* and controls in *black*

Compared to non-CKD control subjects, participants with CKD had lower mean insulin sensitivity (-1.1 (mg/min)/(μU/mL)), higher mean total fat mass (+2.6 kg), and higher mean triglycerides (+53.6 mg/dL) (Table 4). The associations of physical activity with insulin sensitivity and triglycerides did not differ significantly among participants with and without CKD (Fig. 2 and Table 4). However, the association of physical activity with fat mass appeared weaker among participants with CKD, with a *p*-value for interaction that was of borderline statistical significance (*p* = 0.045 without accounting for multiple comparisons).

**Table 4:** Associations of physical activity with metabolic health outcomes in SUGAR, by CKD status

Dependent variable	CKD		Non CKD		
	Mean (SD)^b^	Adjusted difference (95% CI)^a^	Mean (SD)^b^	Adjusted difference (95% CI)^a^	*P* value for Interaction
Insulin sensitivity (mg/min)/(μU/mL)	4.1 (2.1)	0.8 (0.2, 1.4)	5.2 (2.0)	1.0 (0.1, 1.9)	0.73
Fat mass (kg)	32.0 (11.4)	−4.9 (−10.2, 0.4)	29.4 (14.8)	−13.2 (−20.2, −6.2)	0.045
BMI (kg/m^2^)	30.0 (5.5)	−2.0 (−4.5, 0.6)	28.0 (6.8)	−6.3 (−9.4, −3.2)	0.03
CRP (percent difference)	0.3 (2.6)	−11 (−44, 43)	0.1 (2.8)	−41 (−66, 2)	0.26
HDL (mg/dL)	52.4 (22.4)	2.2 (−8.2, 12.6)	58.7 (18.9)	13.9 (5.8, 22.0)	0.07
Triglycerides (mg/dL)	153.2 (91.6)	−39.8 (−90.3, 10.8)	99.6 (66.8)	−30.7 (−57.9, −3.5)	0.76
Systolic BP (mmHg)	134.5 (14.3)	−0.2 (−6.3, 6.0)	122.6 (13.9)	−7.5 (−13.1, −2.0)	0.07
Diastolic BP (mmHg)	79.6 (9.5)	1.3 (−2.4, 5.1)	76.4 (10.1)	−5.2 (−8.5, −1.9)	0.007

^**a**^Difference per doubling of CPM, adjusted for age, sex, race (white/black/other), cardiovascular disease, eGFR. Lipid-lowering medications (statins, fibrates, and niacin medications, included for HDL-cholesterol and triglycerides only), and antihypertensive medications (yes/no and number of antihypertensive medications, for systolic and diastolic BP only), as in Model 2 of Table 3

^b^Entry for CRP is geometric mean (SD)

## Discussion

In this study, we found that higher levels of physical activity, measured using gold standard accelerometry, were associated with greater insulin sensitivity, lower fat mass, and lower serum triglyceride concentration. These associations were independent of demographics, cardiovascular disease, and eGFR and were similar when physical activity was evaluated as average daily movement (CPM) or as proportion of time spent non-sedentary. Participants with CKD tended to have lower physical activity and insulin sensitivity and higher fat mass and serum triglyceride concentrations, compared with healthy control subjects. Nonetheless, associations of physical activity with metabolic health outcomes were generally similar among participants with and without CKD.

Prior studies have shown that patients with CKD are less physically active than the general population, an observation that we also made in our study [23]. Moreover, among people with CKD, greater physical activity has been associated with decreased risks of CKD progression, cardiovascular events, and mortality [24–26]. In the general population, physical activity has been correlated with reduced risks of diabetes, stroke, coronary artery disease, congestive heart failure, and hypertension [14, 23, 24, 27, 28]. In this context, our results help define a plausible biologic basis for the potential clinical benefits of physical activity in CKD. Specifically, our study provides precise, quantitative data demonstrating independent associations of physical activity with major components of metabolic health that may mediate effects of physical activity on long-term clinical outcomes. Our results are complementary to and consistent with those of the cohort studies noted above and with exercise training studies suggesting that increased activity decreases adiposity and inflammation in CKD [29].

Insulin sensitivity is an important measure of metabolic health, with lower sensitivity (insulin resistance) comprising a core component of the metabolic syndrome [30]. This study demonstrated that physical activity is directly correlated with insulin sensitivity, using gold standard measurements of each. The association of physical activity with insulin sensitivity was significant after adjustment for demographic variables, prevalent cardiovascular disease, and eGFR, but somewhat attenuated with further adjustment for fat mass. This observation suggests that the relationship of physical activity with insulin sensitivity is mediated partly, but not wholly, through reduced adiposity. The hyperinsulinemic-euglycemic clamp measures total body insulin sensitivity, which mostly reflects skeletal muscle insulin sensitivity because endogenous glucose production by the liver is nearly or fully suppressed at the insulin infusion rate we applied [4]. It is therefore possible that our observed association of physical activity with insulin sensitivity reflects beneficial effects of exercise on muscle metabolism. It is also possible that insulin sensitivity is influenced by other comorbidities not accounted for in this study. The association of physical activity with insulin sensitivity was significant when evaluated within CKD participants only, and we did not observe an interaction of physical activity with CKD status, suggesting that physical activity may be associated with improving insulin sensitivity.

The inverse association of physical activity with fat mass appeared weaker in participants with versus without CKD. Associations of adiposity with mortality have also been reported to be weaker in people with versus without CKD [31, 32], suggesting that CKD may alter both the causes and consequences of obesity. However, our observed interaction was of borderline statistical significance, particularly considering the fact that we tested multiple potential interactions, and may not represent a true difference by CKD status. Adiposity may promote cardiovascular disease and progression of kidney disease through inflammation and impaired lipoprotein metabolism [33–35]. Therefore, physical activity may reduce adiposity, which may lead to a reduction in cardiovascular disease and progression of kidney disease. Physical activity was also correlated with serum triglyceride concentration. Triglycerides may have direct lipotoxic actions on kidney and vascular tissue and may promote kidney and vascular disease through increased inflammation [36, 37].

We did not observe statistically significant associations of physical activity with inflammation or blood pressure. Other studies have shown inverse correlations of physical ability [27] measured by questionnaire with physical activity [14] to reduced levels of CRP. We may not have shown a significant association because of limited power and the use of only one inflammatory marker. Also, we examined levels of CRP in the blood, while the mechanism of action might be in relevant tissues like adipose and muscle. BP may not have shown correlation due to physical activity being only one of many possible influences affecting BP, making it difficult to find a signal among much noise. We may have been able to detect associations with more precise 24 h ambulatory BP measurements. We observed trends toward expected associations with CRP and BP that may have been statistically significant in a larger study.

Physical activity (CPM) and active time yielded similar metabolic outcome associations, with adjusted HAP yielding weaker associations. This suggests that HAP may misclassify physical activity and that studies that used HAP may have had more impressive associations had physical activity been directly measured by accelerometer. Although physical activity (CPM) and active time yielded similar outcome associations, it is difficult to determine which is more beneficial due to the small study size and the correlation between these two aspects of movement within our study. A larger study has examined the concept in relationship with mortality in greater detail and found a decrease in mortality with increased physical activity and duration of activity [24].

Several factors contribute to the strengths and limitations of our study. Accelerometry is the gold standard for objectively measuring physical activity as it is more precise and less subject to recall bias than questionnaires, which have been used in most prior studies [18, 38]. The hyperinsulinemic-euglycemic clamp is a gold standard method for the measurement of insulin sensitivity and as such is a strength of our study. However, the accelerometer does not account for potential inaccuracies caused by missing data due to patients not wearing the devices. Also the wear period may not accurately reflect a patient’s extended physical activity. Our study is further strengthened by our examination of a clinic-based population of importance to practicing nephrologists and the evaluation of a comprehensive set of metabolic outcomes that may be key mediators of cardiovascular and renal risk in CKD patients. The cross-sectional design of the study limits our ability to ascertain long-term clinical outcomes or discern causal relationships. The modest size of our study may increase the influence of outliers, reduce statistical power, and increase the chance that factors important to this population were missed.

## Conclusion

In conclusion, this study suggests that greater physical activity is associated with improved metabolic health for patients with moderate-severe CKD by increasing insulin sensitivity and reducing adiposity and serum triglycerides. This study identifies insulin sensitivity, adiposity, and dyslipidemia as logical intermediate targets for short-term physical activity trials that assess what types of physical activity may best promote metabolic health in CKD. Larger studies are needed to examine the long term effects of physical activity and its potential health benefits for patients with CKD.

## Additional file

Additional file 1: Supplementary Material Physical Activity and Metabolic Health in CKD. **Figure S1** and **Tables S1–5**). (DOCX 63 kb)

## Abbreviations

AER: Albumin excretion rate
BP: Blood pressure
CKD: Chronic kidney disease
CPM: Counts per minute
CRP: C-reactive protein
CVD: Cardiovascular disease
eGFR: Estimated glomerular filtration rate
HAP: Human activity profile
SD: Standard deviation
SI: Insulin sensitivity
SUGAR: Study of glucose and insulin in renal disease
