# Supplementary material for: Physical activity and metabolic health in chronic kidney disease: a cross-sectional study
Source: BMC Nephrol. 2016 Nov 22;17:187. doi: 10.1186/s12882-016-0400-x (PMC5120456; doi:10.1186/s12882-016-0400-x)
Supplement: Supplementary file 1 — Supplementary Material Physical Activity and Metabolic Health in CKD. Figure S1 and Tables S1–5). (DOCX 63 kb) [file 12882_2016_400_MOESM1_ESM.docx]

**Additional file for:**

**Physical activity and metabolic health in chronic kidney disease: a cross-sectional study**

**Authors:** Wilson Bowlby, BS, Leila Zelnick, PhD, Connor Henry, BA, Jonathan Himmelfarb, MD, Steven E. Kahn, MB, ChB, Bryan Kestenbaum, MD, MS, Cassianne Robinson-Cohen, PhD, Kristina Utzschneider, MD, Ian H. de Boer, MD, MS

**Table S1. Characteristics of participants in the Study of Glucose and Renal Disease (SUGAR) who were included or excluded from the current analysis**

|  | **Included** | **Excluded** |
| --- | --- | --- |
| N | 76 | 22 |
| **Demographics:** |  |  |
| Age (years) | 63.5 (12.2) | 59.5 (16.4) |
| Female sex | 34 (45) | 13 (59) |
| Race |  |  |
| White | 61 (80) | 14 (64) |
| Black | 11 (14) | 6 (27) |
| Other | 4 (5) | 2 (9) |
| **Medical history & lifestyle:** |  |  |
| Cardiovascular disease | 18 (24) | 3 (14) |
| Current smoking | 11 (14) | 2 (9) |
| Physical activity (adjusted activity score), median | 74.5 (66.8-81.0) | 73.0 (65.5-82.8) |
| Subjects with CKD | 47 (62) | 12 (55) |
| **Medication use:** |  |  |
| Any antihypertensive medication | 52 (68) | 14 (64) |
| Number of antihypertensive medications | 1.7 (1.6) | 1.1 (1.2) |
| Any lipid-lowering medication | 24 (32) | 8 (36) |
| Statin | 21 (28) | 7 (32) |
| Fibrate | 5 (7) | 1 (5) |
| Niacin | 3 (4) | 1 (5) |
| **Physical characteristics:** |  |  |
| Height (cm) | 172.2 (10.4) | 168.8 (10.7) |
| Weight (kg) | 86.8 (19.7) | 82.7 (22.1) |
| Fat mass (kg) | 31.0 (12.8) | 27.5 (12.4) |
| BMI (kg/m^2^) | 29.2 (6.0) | 28.9 (7.1) |
| Systolic blood pressure (mm Hg) | 129.9 (15.2) | 120.6 (10.6) |
| Diastolic blood pressure (mm Hg) | 78.4 (9.8) | 77.1 (7.8) |
| **Laboratory data:** |  |  |
| Serum creatinine (mg/dL), median | 1.5 (0.9-1.8) | 1.3 (0.9-2.0) |
| Serum cystatin C (mg/L), median | 1.3 (1.0-1.6) | 1.3 (0.8-1.9) |
| Estimated GFR (mL/min/1.73 m^2^) | 57.2 (27.7) | 60.4 (33.6) |
| Urine AER, (mg/24 hours) median | 12.7 (5.9-96.5) | 16.9 (5.1-97.9) |

Mean (SD) presented for continuous variables, and median (IQR) as noted; N (%) presented for all categorical variables

**Table S2. Characteristics of analysis population by CKD status**

|  | **eGFR ≥ 60 mL/min/1.73m^2^** | **eGFR < 60 mL/min/1.73m^2^** |
| --- | --- | --- |
| N | 29 | 47 |
| **Demographics:** |  |  |
| Age (years) | 60.8 (11.0) | 65.1 (12.8) |
| Female sex | 13 (45) | 21 (45) |
| Race |  |  |
| White | 27 (93) | 34 (72) |
| Black | 1 (3) | 10 (21) |
| Other | 1 (3) | 3 (6) |
| **Medical history & lifestyle:** |  |  |
| Cardiovascular disease | 2 (7) | 16 (34) |
| Current smoking | 2 (7) | 9 (19) |
| Accelerometry data |  |  |
| Sedentary time (%) | 57.7 (12.6) | 64.9 (12.2) |
| Light activity time (%) | 33.6 (9.3) | 29.5 (9.2) |
| Moderate activity time (%) | 8.7 (5.3) | 5.7 (4.8) |
| Questionnaire data |  |  |
| Adjusted activity score, median | 79.0 (74.0-84.0) | 71.0 (61.5-78.5) |
| Maximum HAP score | 83.7 (6.6) | 76.9 (9.9) |
| **Medication use:** |  |  |
| Any antihypertensive medication | 10 (34) | 42 (89) |
| Number of antihypertensive medications | 0.4 (0.6) | 2.4 (1.6) |
| Any lipid-lowering medication | 3 (10) | 21 (45) |
| Statin | 2 (7) | 19 (40) |
| Fibrate | 1 (3) | 4 (9) |
| Niacin | 1 (3) | 2 (4) |
| **Physical characteristics:** |  |  |
| Height (cm) | 173.1 (10.3) | 171.6 (10.5) |
| Weight (kg) | 84.0 (20.6) | 88.6 (19.1) |
| Fat mass (kg) | 29.4 (14.8) | 32.0 (11.4) |
| BMI | 28.0 (6.8) | 30.0 (5.5) |
| Systolic blood pressure (mmHg) | 122.6 (13.9) | 134.5 (14.3) |
| Diastolic blood pressure (mmHg) | 76.4 (10.1) | 79.6 (9.5) |
| **Laboratory data:** |  |  |
| Serum creatinine (mg/dL), median | 0.9 (0.7-1.0) | 1.7 (1.5-2.0) |
| Serum cystatin C (mg/L), median | 0.9 (0.8-1.0) | 1.6 (1.3-1.9) |
| Estimated GFR (mL/min/1.73 m^2^) | 87.2 (16.7) | 38.7 (12.9) |
| Urine AER, (mg/24 hours) median | 6.0 (4.1-9.1) | 39.2 (12.2-198.9) |

Mean (SD) presented for continuous variables, and median (IQR) as noted; N (%) presented for all categorical variables

**Table S3. Adjusted associations of percent time active (measured by accelerometry) with metabolic health outcomes**

|  | **Unadjusted** | **Model 1** | **Model 2** | **Model 3** |
| --- | --- | --- | --- | --- |
| **Outcomes:** |  |  |  |  |
| Insulin sensitivity (mg/min)/(µU/mL) | 0.6 (0.2, 1.0) | 0.5 (0.1, 0.9) | 0.5 (0.2, 0.9) | 0.5 (0.1, 0.9) |
| *P Value* | 0.001 | 0.007 | 0.005 | 0.03 |
| Fat Mass (kg) | -3.3 (-5.5, -1.0) | -4.5 (-7.0, -2.0) | -4.2 (-7.0, -1.5) | NA |
| *P Value* | 0.005 | 0.0004 | 0.002 | NA |
| BMI (kg/m^2^) | -1.3 (-2.4, -0.2) | -2.1 (-3.2, -0.9) | -2.0 (-3.3, -0.7) | -0.3 (-0.9, 0.4) |
| *P Value* | 0.02 | 0.0003 | 0.002 | 0.45 |
| CRP (% difference) | -18 (-30, -3) | -19 (-32, -5) | -14 (-30, 5) | -4 (-23, 21) |
| *P Value* | 0.02 | 0.01 | 0.14 | 0.74 |
| HDL (mg/dL) | 3.2 (0.0, 6.4) | 2.3 (-2.7, 7.2) | 2.2 (-2.2, 6.6) | 1.4 (-2.9, 5.7) |
| *P Value* | 0.051 | 0.37 | 0.33 | 0.53 |
| Triglycerides (mg/dL) | -23.0 (-38.7, -7.3) | -26.3 (-42.2, -10.4) | -22.4 (-39.5, -5.3) | -14.6 (-30.7, 1.6) |
| *P Value* | 0.004 | 0.001 | 0.01 | 0.08 |
| Systolic BP (mm Hg) | -2.5 (-5.2, 0.1) | -2.7 (-5.3, -0.2) | -1.9 (-4.7, 0.9) | -0.2 (-3.0, 2.5) |
| *P Value* | 0.06 | 0.04 | 0.19 | 0.87 |
| Diastolic BP (mm Hg) | 0.1 (-1.7, 1.9) | -1.3 (-2.8, 0.3) | -1.3 (-2.9, 0.4) | -1.0 (-2.8, 0.7) |
| *P Value* | 0.89 | 0.12 | 0.13 | 0.25 |

*****Entries are the difference (95% CI) in the outcome associated with a +10 difference in percent time active, except as noted. Model 1 adjusts for age, sex, and race (white/black/other). Model 2 additionally adjusts for cardiovascular disease and eGFR. For HDL/Triglycerides/LogTriglycerides outcomes, Model 2 additionally adjusts for statins, fibrates, and niacin medications; for Systolic BP (mmHg)/Diastolic BP (mmHg) outcomes, Model 2 additionally adjusts for the number of hypertension medications. Model 3 adjusts for Fat Mass (Kg), where applicable.

**Table S4. Multivariable associations of percent time spent in moderate-vigorous activity with metabolic health outcomes**

|  | **Unadjusted** | **Model 1** | **Model 2** | **Model 3** |
| --- | --- | --- | --- | --- |
| **Outcomes:** |  |  |  |  |
| Insulin sensitivity (mg/min)/(µU/mL) | 2.9 (1.3, 4.4) | 3.7 (1.9, 5.4) | 3.5 (1.9, 5.2) | 1.7 (0.2, 3.2) |
| *P Value* | 0.0003 | < 0.0001 | < 0.0001 | 0.02 |
| Fat Mass (Kg) | 1.6 (0.7, 2.4) | 1.5 (0.6, 2.3) | 1.5 (0.6, 2.5) | 1.4 (0.3, 2.4) |
| *P Value* | 0.0002 | 0.001 | 0.001 | 0.009 |
| BMI | -10.2 (-15.8, -4.7) | -13.9 (-20.0, -7.8) | -14.1 (-20.7, -7.5) | NA |
| *P Value* | 0.0003 | < 0.0001 | < 0.0001 | NA |
| CRP (% difference) | 7.4 (3.4, 11.3) | 6.2 (2.8, 9.6) | 3.6 (0.6, 6.6) | 2.0 (-1.4, 5.3) |
| *P Value* | 0.0003 | 0.0004 | 0.02 | 0.25 |
| HDL (mg/dL) | -31 (-56, 8) | -26 (-56, 24) | -16 (-50, 43) | 32 (-20, 120) |
| *P Value* | 0.10 | 0.25 | 0.53 | 0.28 |
| Triglycerides (mg/dL) | 6.7 (-3.3, 16.7) | 10.3 (-0.2, 20.7) | 10.1 (1.6, 18.6) | 8 (-0.6, 16.6) |
| *P Value* | 0.19 | 0.054 | 0.02 | 0.07 |
| Systolic BP (mmHg) | -46.4 (-85.6, -7.1) | -55.1 (-90.6, -19.5) | -47.3 (-94.4, -0.3) | -17.2 (-65.1, 30.7) |
| *P Value* | 0.02 | 0.002 | 0.049 | 0.48 |
| Diastolic BP (mmHg) | -0.4 (-0.6, -0.1) | -0.5 (-0.7, -0.2) | -0.4 (-0.7, -0.1) | -0.2 (-0.5, 0.1) |
| *P Value* | 0.004 | 0.0001 | 0.006 | 0.22 |

*****Entries are the difference (95% CI) in the outcome associated with spending ≥ 5% of time in moderate activity, compared with spending <5% in moderate activity, except as noted. Model 1 adjusts for age, sex, and race (white/black/other). Model 2 additionally adjusts for cardiovascular disease and eGFR. For HDL/Triglycerides outcomes, Model 2 additionally adjusts for statins, fibrates, and niacin medications; for Systolic BP (mmHg)/Diastolic BP (mmHg) outcomes, Model 2 additionally adjusts for the number of hypertension medications. Model 3 adjusts for Fat Mass (Kg), where applicable.

**Table S5. Multivariable associations of percent time spent in moderate-vigorous activity with metabolic health outcomes**

|  | **Unadjusted** | **Model 1** | **Model 2** | **Model 3** |
| --- | --- | --- | --- | --- |
| **Outcomes:** |  |  |  |  |
| Insulin sensitivity (mg/min)/(µU/mL) | 1.3 (0.1, 2.4) | 1.5 (0.3, 2.7) | 1.3 (0.1, 2.5) | 0.7 (-0.5, 1.8) |
| *P Value* | 0.03 | 0.02 | 0.03 | 0.25 |
| Fat Mass (Kg) | 0.7 (0.3, 1.1) | 0.6 (0.2, 1.0) | 0.7 (0.2, 1.1) | 0.6 (0.1, 1.0) |
| *P Value* | 0.0003 | 0.003 | 0.004 | 0.02 |
| BMI | -4.0 (-6.6, -1.3) | -5.0 (-7.8, -2.1) | -4.7 (-7.8, -1.6) | NA |
| *P Value* | 0.003 | 0.0007 | 0.003 | NA |
| Human Activity Profile (HAP) | -1.9 (-3.2, -0.7) | -2.4 (-3.7, -1.2) | -2.3 (-3.7, -0.9) | -0.4 (-1.1, 0.3) |
| *P Value* | 0.003 | 0.0002 | 0.001 | 0.26 |
| CRP (% difference) | 2.7 (0.8, 4.5) | 2.1 (0.6, 3.6) | 1.1 (-0.6, 2.7) | 0.4 (-1.3, 2.1) |
| *P Value* | 0.006 | 0.006 | 0.20 | 0.62 |
| HDL (mg/dL) | -20 (-37, 1) | -19 (-36, 4) | -12 (-33, 16) | 0 (-24, 33) |
| *P Value* | 0.06 | 0.10 | 0.36 | 0.98 |
| Triglycerides (mg/dL) | 3.5 (-1.4, 8.4) | 4.4 (-0.8, 9.6) | 4.6 (0.2, 9) | 3.8 (-0.3, 7.8) |
| *P Value* | 0.16 | 0.10 | 0.04 | 0.07 |
| Systolic BP (mmHg) | -25.1 (-41.9, -8.2) | -27.4 (-42.3, -12.6) | -21.4 (-38.5, -4.4) | -11.6 (-27.7, 4.5) |
| *P Value* | 0.004 | 0.0003 | 0.01 | 0.16 |
| Diastolic BP (mmHg) | -0.2 (-0.3, -0.1) | -0.2 (-0.3, -0.1) | -0.2 (-0.3, 0.0) | -0.1 (-0.2, 0.0) |
| *P Value* | 0.0009 | 0.0001 | 0.008 | 0.14 |

*****Entries are the difference (95% CI) in the outcome associated with a +5 difference in percent time spent in moderate activity, except as noted. Model 1 adjusts for age, sex, and race (white/black/other). Model 2 additionally adjusts for cardiovascular disease and eGFR. For HDL/Triglycerides/LogTriglycerides outcomes, Model 2 additionally adjusts for statins, fibrates, and niacin medications; for Systolic BP (mmHg)/Diastolic BP (mmHg) outcomes, Model 2 additionally adjusts for the number of hypertension medications. Model 3 adjusts for Fat Mass (Kg), where applicable.

**Table S6. Adjusted associations of adjusted activity score (ascertained by questionnaire) with metabolic health outcomes**

|  | **Unadjusted** | **Model 1** | **Model 2** | **Model 3** |
| --- | --- | --- | --- | --- |
| **Outcomes:** |  |  |  |  |
| Insulin sensitivity (mg/min)/(µU/mL) | 0.4 (0.0, 0.7) | 0.3 (-0.1, 0.8) | 0.3 (-0.2, 0.8) | 0.2 (-0.3, 0.8) |
| *P Value* | 0.03 | 0.13 | 0.19 | 0.36 |
| Fat Mass (kg) | -2.4 (-4.6, -0.2) | -3.3 (-5.7, -1.0) | -3.2 (-5.6, -0.8) | NA |
| *P Value* | 0.03 | 0.006 | 0.01 | NA |
| BMI (kg/m^2^) | -1.0(-2.0, 0.0) | -1.4 (-2.4, -0.3) | -1.3 (-2.4, -0.3) | 0.0 (-0.7, 0.7) |
| *P Value* | 0.06 | 0.01 | 0.01 | 0.99 |
| CRP (% difference) | -15 (-27, 0) | -14 (-28, 2) | -13 (-27, 3) | -5 (-21, 14) |
| *P Value* | 0.04 | 0.09 | 0.12 | 0.57 |
| HDL (mg/dL) | -3.3 (-10.9, 4.4) | -2.3 (-9.8, 5.2) | -4.3 (-12.0, 3.4) | -5.5 (-13.2, 2.1) |
| *P Value* | 0.40 | 0.55 | 0.27 | 0.16 |
| Triglycerides (mg/dL) | -10.1 (-24.6, 4.5) | -14.5 (-29.8, 0.8) | -14.2 (-35.6, 7.3) | -7.1 (-29.9, 15.7) |
| *P Value* | 0.17 | 0.06 | 0.20 | 0.54 |
| Systolic BP (mm Hg) | -2.9 (-5.0, -0.7) | -1.5 (-3.8, 0.8) | -1.5 (-3.7, 0.7) | -0.3 (-2.5, 1.8) |
| *P Value* | 0.009 | 0.21 | 0.18 | 0.76 |
| Diastolic BP (mm Hg) | 0.1 (-1.5, 1.7) | 0.3 (-1.2, 1.7) | 0.2 (-1.3, 1.7) | 0.5 (-1.0, 2.0) |
| *P Value* | 0.89 | 0.71 | 0.79 | 0.51 |

*****Entries are the difference (95% CI) in the outcome associated with a +10 difference in adjusted activity score, except as noted. Model 1 adjusts for age, sex, and race (white/black/other). Model 2 additionally adjusts for cardiovascular disease and eGFR. For HDL/Triglycerides/LogTriglycerides outcomes, Model 2 additionally adjusts for statins, fibrates, and niacin medications; for Systolic BP (mmHg)/Diastolic BP (mmHg) outcomes, Model 2 additionally adjusts for the number of hypertension medications. Model 3 adjusts for Fat Mass (Kg), where applicable.

**Figure S1. Flow diagram of participant enrollment and analysis.**
